# Functional Bowel Disorders Are Associated with a Central Immune Activation

**DOI:** 10.1155/2017/1642912

**Published:** 2017-10-23

**Authors:** Per G. Farup, Thor Ueland, Knut Rudi, Stian Lydersen, Knut Hestad

**Affiliations:** ^1^Department of Research, Innlandet Hospital Trust, 2381 Brumunddal, Norway; ^2^Unit for Applied Clinical Research, Department of Clinical and Molecular Medicine, Faculty of Medicine and Health Sciences, Norwegian University of Science and Technology, 7491 Trondheim, Norway; ^3^Research Institute of Internal Medicine, Oslo University Hospital, Oslo, Norway; ^4^Department of Chemistry, Biotechnology and Food Science, Norwegian University of Life Sciences, P.O. Box 5003, 1432 Ås, Norway; ^5^Regional Centre for Child and Youth Mental Health and Child Welfare, Faculty of Medicine and Health Sciences, Norwegian University of Science and Technology, 7491 Trondheim, Norway; ^6^Department of Psychology, Faculty of Social Sciences and Technology Management, Norwegian University of Science and Technology, 7491 Trondheim, Norway; ^7^Inland Norway University of Applied Sciences, 2381 Elverum, Norway

## Abstract

**Background:**

Subjects with depression and unexplained neurological symptoms have a high prevalence of gastrointestinal comorbidity probably related to the brain-gut communication. This study explored associations between functional gastrointestinal disorders (FGID) and inflammatory markers in subjects with these disorders.

**Methods:**

The FGID, including irritable bowel syndrome (IBS), were classified according to the Rome III criteria, and degree of symptoms was assessed with IBS symptom severity score (IBS-SSS). A range of interleukins (IL), chemokines and growth factors, tryptophan, and kynurenine were analysed in serum and the cerebrospinal fluid (CSF), and short-chain fatty acids (SCFA) were analysed in the faeces. The results are reported as partial correlation (pc) and *p* values.

**Results:**

Sixty-six subjects were included. IBS was associated with high levels of tryptophan (*p* = 0.048) and kynurenine (*p* = 0.019) and low level of IL-10 (*p* = 0.047) in the CSF. IBS-SSS was associated with high tumor necrosis factor and low IL-10 in the CSF; pc = 0.341 and *p* = 0.009 and pc = −0.299 and *p* = 0.023, respectively. Propionic minus butyric acid in faeces was negatively associated with IL-10 in the CSF (pc = −0.416, *p* = 0.005).

**Conclusions:**

FGID were associated with a proinflammatory immune activation in the central nervous system and a disturbed tryptophan metabolism that could have been mediated by the faecal microbiota.

## 1. Background

Comorbidities are common in subjects with depression and subjects with unexplained somatic symptoms [1, 2]. Functional gastrointestinal disorders (FGID) are particularly common, probably due to the cross talk between the brain and the gut through the brain-gut axis [3]. Irritable bowel syndrome (IBS) is a common functional gastrointestinal disorder (FGID) with a prevalence rate of approximately 8% in the Western world [4]. The prevalence rates are significantly higher in subjects with depression and unexplained somatic disorders [1, 2]. IBS is characterised by abdominal pain and disturbed bowel functions and a high prevalence of comorbidities such as muscle-skeletal pain, fatigue, anxiety, and depression [5, 6]. The syndrome links the mind and the body and has been described as a biopsychosocial disorder [7]. This term focuses on the bidirectional relations between the mind and the body and puts aside the view of diseases as either somatic or psychological. The cross talk between the mind and the gut passes through the brain-gut axis that involves the gut microbiota and the neuronal, neuroendocrine, and immunological system [8, 9]. IBS and depression have both been associated with immune alterations and faecal dysbiosis [10–13]. The local immune activation seen in subjects with IBS is modulated by the gut microbiota and alters the central neural functioning [14]. Faecal dysbiosis could be a mediator or confounder in the brain-gut communication. Whether the microbiota per se or the microbiota's metabolic activity such as the production of short-chain fatty acids (SCFA) is involved in the communication is partly unknown [14]. The communication could be “bottom-up” (the gut is the cause of the symptoms from the central nervous system) or “top-down” (the central nervous system is the cause of the gastrointestinal symptoms) depending on the primary alterations [15].

Depression and FGID have several common pathophysiological abnormalities. Both disorders have been associated with neural, neuroendocrine, and immune disorders and deviant tryptophan, serotonin, and kynurenine metabolism [3, 10, 16–18]. The role of the tryptophan, serotonin, and kynurenine metabolism has been attributed to be of great importance [19–21]. Others have focused on the “immune hypothesis” and studied immunological markers in serum and the colonic mucosa [10, 16, 22, 23]. Cytokines and neurotrophic factors in the cerebrospinal fluid (CSF) have been studied in various chronic pain disorders, but not in IBS [24]. The difference between IBS and most of the studied pain disorders is that the aetiology and pathophysiology of IBS are unknown. The subjects included in this study had symptoms related to the central nervous system and a high prevalence of gastrointestinal comorbidity and were suitable for the study of the gut-brain interaction.

The aims of this study were to relate the presence of IBS and the degree of gastrointestinal complaints to the tryptophan metabolism and inflammatory markers in the blood, the CSF, and faeces in subjects with depression and unexplained neurological symptoms.

## 2. Methods

### 2.1. Study Design

Two groups of subjects, one with depression and one with unexplained neurological symptoms, were included in this cross-sectional study. The primary aim of this study was a secondary aim of a larger project [25]. The subjects were recruited from the psychiatric and neurological departments at Innlandet Hospital Trust, Norway. To ascertain the diagnoses of depression and unexplained neurological symptoms and exclude other somatic disorders, the subjects in the depression group were evaluated by a clinical psychologist and those in the neurological group by a senior consultant in neurology. The participants filled in paper-based questionnaires. A trained study nurse was responsible for the patient compliance and the practical work. Biomarkers in the blood, CSF, and faeces were compared in subjects with and without IBS and associated with the degree of abdominal complaints.

### 2.2. Participants

The study included consecutive subjects above 18 years of age with a diagnosis of idiopathic depression according to ICD-10, F 32-34 spectre (the depression group), and subjects with neurological symptoms that remained unexplained after thorough examinations (the neurological group). In all subjects, a detailed medical history and comprehensive clinical, laboratory, and other investigations were performed according to the doctors' discretion. Subjects with drug or alcohol abuse or organic diseases were excluded. Gender, age (years), previous and present disorders, and use of medicine were noted, and a standard clinical and neurological examination was performed.

### 2.3. Variables

#### 2.3.1. Depression

For depression, the following are used: Beck Depression Inventory-II (BDI-II) (symptoms: minimal, mild, moderate, and severe depression; scores: 0–13, 14–19, 20–28, and 29–63, resp.) and Montgomery-Åsberg Depression Scale (MADRS) (symptoms: absent, mild, moderate, and severe depression; scores: 0–6, 7–19, 20–34, and 35–60, resp.).

#### 2.3.2. Abdominal Complaints

IBS (yes/no) was determined according to the Rome III criteria. The subjects filled in a validated Norwegian translation of the Rome III questionnaire on a paper. A gastroenterologist (the author PGF) performed the scoring.

IBS symptom severity score (IBS-SSS), which has been recommended by the Rome Foundation, was used to score the severity of abdominal complaints [26]. The questionnaire was, however, responded to by all subjects and not only subjects with IBS and was used as a measure of the bowel symptom severity independent of the presence of IBS. The score ranges from 0 to 500.

#### 2.3.3. Blood Tests

The blood samples were collected in the morning before 10 a.m., were allowed to clot for 30 minutes, were centrifuged for 10 minutes at 2000*g*, and were stored at −70°C before being analysed. The subjects were not asked to be fasting.

Twenty-seven interleukins (IL), chemokines, and growth factors were analysed in serum: IL-1*β*; IL-1 receptor antagonist (IL-1Ra); IL-2; IL-4; IL-5; IL-6; IL-7; IL-8 (CXCL8); IL-9; IL-10; IL-12 (p70); IL-13; IL-15; IL-17; eotaxin/CCL11; basic fibroblast growth factor; granulocyte-colony stimulating factor (G-CSF); granulocyte macrophage CSF; interferon- (IFN-) *γ*; IFN-inducible protein 10 (IP-10; CXCL10); monocyte chemoattractant protein 1 (MCP-1; CCL2); macrophage inflammatory peptide- (MIP-) 1*α* (CCL3); MIP-1*β* (CCL4); platelet-derived growth factor-BB; regulated on activation, normal T-cell expressed and secreted (RANTES; CCL5) tumor necrosis factor (TNF); and vascular endothelial growth factor. The samples were analysed on a Multiplex Analyzer using a multiplex cytokine assay (Bio-Plex Human Cytokine 27-Plex Panel; Bio-Rad Laboratories Inc., Hercules, CA, USA). The TNF/IL-10 ratio was calculated because the results indicated an inverse correlation.

Tryptophan, kynurenine, and kynurenine/tryptophan ratio × 1000 (K/T ratio × 1000) were measured in serum. Free tryptophan and kynurenine were extracted by the addition of trichloroacetic acid followed by centrifugation. The supernatant was then analysed using gradient elution and quantified by ultraviolet (UV) for kynurenine and tryptophan. The LC system was of type Infinity 1290 (Agilent Technologies Inc., Palo Alto, CA, USA) applying a reversed phase Kinetex, 2.6 *μ* C18 100A UPLC column (Phenomenex). The measurements were performed by Vitas AS, Oslo, Norway (tryptophan: range 10–100 *μ*M; kynurenine: range 0.8–5 *μ*M; detection limit for both: 0.1 *μ*M).

#### 2.3.4. Cerebrospinal Fluid (CFS)

Spinal puncture was performed on nonfasting subjects before 12 a.m., except for two subjects who had the puncture at 1 p.m. The spinal fluid was immediately stored at −70°C.

Twenty-four ILs, chemokines, and growth factors (the same ones as in serum except for IL-7, IL-12, and G-CSF that were not detectable in the CSF) and tryptophan, kynurenine, and K/T ratio × 1000 were analysed in the CSF.

#### 2.3.5. Faecal Samples

Stool collected at home was immediately frozen at −20°C and later transported to the hospital in the frozen state. The stool collected at the hospital and the stool transported to the hospital at −20°C were immediately brought to a freezer with −70°C. Within one month, S.T.A.R. (Stool Transport and Recovery; Roche, Basel, Switzerland) buffer solution was added to the samples (stool/S.T.A.R. ratio 1/3) and vortexed to a homogenous suspension and stored at −80°C.

Stool samples were analysed for short-chain fatty acids (acetic acid, propionic acid, butyric acid, isobutyric acid, valeric acid, and isovaleric acid) by gas chromatography. The Thermo Focus with a flame ionization detector (Thermo Fisher, Waltham, Massachusetts, USA) was used. Helium was used as carrier gas, and the column used was 30 m-long Stabilwax-DA from Restek with polyethylene glycol (PEG) as the stationary phase. The chromatography conditions were as follows: gas pressure 0.6 bar; injector temperature 210°C; start temperature 80°C; temperature program 0°C/min for 3 min, 23.3°C/min for 3 min, and 25°C/min for 2 min; end temperature 200°C; and detector temperature 230°C [27]. In addition to the individual fatty acids, the difference between propionic and butyric acids (PmB), which has been shown to be a potential biomarker for IBS, was used in the analyses [28].

#### 2.3.6. Supplementary Investigations

In the neurological group, computer tomography or magnetic resonance imaging was performed in all subjects. Otherwise, supplementary examinations were carried out at the doctors' discretion in both groups.

### 2.4. Statistics

The subjects' characteristics have been reported separately for the neurological and depression groups with comparisons between the groups. All the subsequent regression analyses were adjusted for the groups (depression versus neurological) and the degree of depression, which was found to be a confounder. Since BDI-II was a stronger predictor of IBS and IBS-SSS than MADRS, and BDI-II and MADRS were highly correlated, only BDI-II was used in the analyses. Logistic regression analyses with IBS as the dependent variable and linear regression with IBS-SSS as the dependent variable were carried out with one by one of all the other variables (clinical data, questionnaires, blood tests, analyses of the CSF, and faecal samples) as covariates. From these analyses, we report all the statistically significant covariates as well as the covariates judged as relevant. Variables associated with IBS and IBS-SSS in the first set of analyses were together with the group and depression included in further logistic and linear regression analyses. The analyses have been checked for collinearity, and the residuals have been checked for normality and outliers. IBM SPSS Statistics for Windows, version 22.0. Armonk, NY, IBM Corp., was used for most of the analyses. Fractional polynomial modeling with STATA Release 13, StataCorp LP, Texas, USA, was used for the multivariable analyses where a linear function of the covariate was unfit. *p* values < 0.05 were judged as statistically significant.

### 2.5. Ethics

The study was approved by the Norwegian Regional Committees for Medical and Health Research Ethics, PB 1130, Blindern, 0318 Oslo, Norway (reference number 2009/2196a) and performed in accordance with the Declaration of Helsinki. Written informed consent was given by all participants before inclusion.

## 3. Results

Out of 21 and 49 subjects included in the depression and neurological groups of the main project, 19 and 47, respectively, had serum samples for the analyses of inflammatory markers and satisfactorily filled in questionnaires for the scoring of IBS and were included in this study. Analysis of IBS-SSS was missing in one patient in the neurological group, analyses of the CSF were missing in four subjects in the depression group, and analyses of faeces were missing in two subjects in the neurological group and 14 in the depression group. In the neurological group, fatigue, headache, muscle or other pain syndromes, dizziness, and ataxia were the most commonly reported unexplained symptoms.  Table 1 gives the subjects' characteristics in the two groups with comparisons between the groups.

Table 2 gives the predictors of IBS adjusted for the group and depression (BDI-II). Low values of IL-10 and high values of kynurenine and tryptophan in the CSF and high levels of propionic acid and PmB in faeces were significant predictors of IBS.

Table 3 gives the predictors of IBS-SSS adjusted for the group and depression (BDI-II). High level of TNF and a high TNF/IL-10 ratio in the CSF were predictors of IBS-SSS.

**Figure 1:**
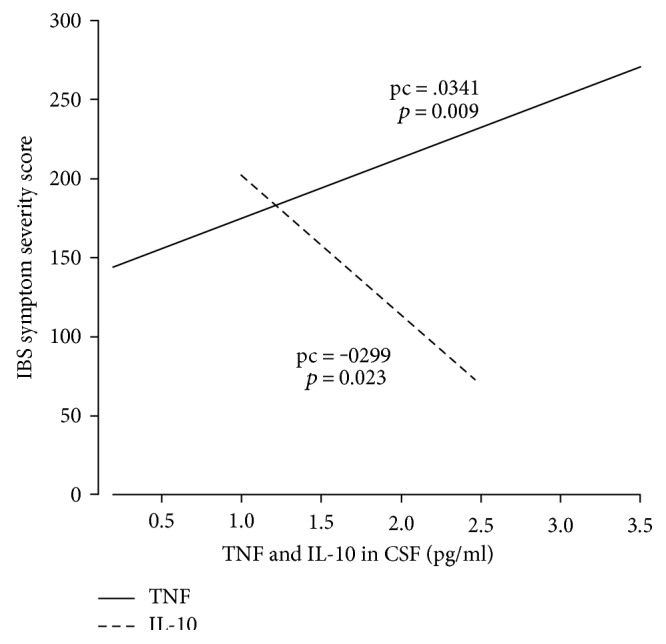
The figure shows the associations between TNF and IL-10 in the cerebrospinal fluid (CSF) and perceived symptom severity (IBS symptom severity score) measured at the average of the other covariates.

Table 4 gives the predictors of IBS and IBS-SSS adjusted for the group, BDI-II, TNF, and IL-10 in serum and CSF.  Figure 1() presents the associations between IBS-SSS and high TNF and low IL-10-CSF values, which were the main findings. PmB was negatively associated with IL-10-CSF after adjusting for the group, BDI-II, and TNF-CSF (B: −0.86; 95% CI: −1.44 to −0.27; *p* = 0.005; pc = −0.416). None of the markers in serum was associated with either IBS or IBS-SSS.

## 4. Discussion

### 4.1. General Discussion

The study confirmed the high prevalence of gastrointestinal comorbidity in subjects with depression and unexplained neurological symptoms and showed significant correlations between FGID and neuroimmune activation and disturbed tryptophan metabolism in the central nervous system. IBS and IBS-SSS were associated with low levels of the anti-inflammatory cytokine IL-10 in the CSF. In addition, IBS-SSS was associated with high levels of the proinflammatory cytokine TNF. The severity of abdominal pain or discomfort (IBS-SSS) and not the cause of the symptoms (IBS) was the main predictor of the proinflammatory response seen in the CSF. It is known that pain, including gastrointestinal pain, involves peripheral neuroimmune reactions, but central neuroimmune reactions have been less well studied. In a published review, Bjurstrom et al. wrote that “*In various preclinical chronic pain models, cytokines and neurotrophic factors have been identified as pivotal mediators involved in neuroimmune activation pathways and cascades, and in neuron-glia interactions. Research confirming these findings in humans has so far been scarce and never evaluated in a comprehensive manner*” [24]. This study supports the preclinical data in a clinical study.

TNF is a pronociceptive cytokine thought to sensitise neurons and stimulate the release of other proinflammatory cytokines such as IL-1*β* and IL6. IL-10 is an anti-inflammatory cytokine acting by inhibition of nuclear factor kappa B and suppressing the production of proinflammatory cytokines including TNF [24]. An increase in TNF and decrease in IL-10 may reflect a net proinflammatory effect in the central nervous system. The associations between the cytokines and IBS-SSS were stronger and more convincing than the associations with IBS, indicating that TNF and IL-10 are better biomarkers of perceived severity of FGID than the presence of IBS.

The composition and function of the faecal microbiota are essential for the immune system and are crucial for the brain-gut communication [14]. Faecal SCFA, which are produced by the microbiota through fermentation of indigestible polysaccharides and proteins, influence gastrointestinal motility, the gastrointestinal immune system, metabolism, and cell proliferation [29, 30]. In contrast to a comprehensive literature on the peripheral effects of the microbiota and SCFA, little is known about the central nervous effects of SCFA. In this study, propionic acid and PmB were significantly associated with IBS, and PmB was negatively associated with IL-10 in the CSF, which may suggest that the SCFA are involved in the brain-gut communication. The SCFA were not significantly associated with the symptom severity. The discrepancy between IBS and perceived symptom severity could indicate that the pathophysiology of IBS differs from that of the perceived severity of the FGID.

Advanced neuroimaging techniques have demonstrated the cerebral involvement in IBS [17, 31, 32]. These findings have not been related to central immunological markers. Preclinical studies implicate that proinflammatory peripheral and central cytokines induce and maintain chronic pain states by central sensitisation [24, 33, 34]. FGID differ from other pain syndromes and preclinical studies by the absence of known aetiology. The central neuroimmune activation shown in this study might be associated with a central sensitisation that contributes to the chronicity of the disorder by an exaggerated and long-lasting response to normal and abnormal stimuli.

In subjects with IBS, a cytokine imbalance in serum and the gastrointestinal mucosa has been shown in several studies [22, 23, 35]. Reviews have demonstrated an imbalance of proinflammatory TNF and anti-inflammatory IL-10 in serum/plasma of subjects with IBS. [23, 36]. This study could not confirm the results, but the same cytokines in the CSF were associated with symptom severity. In most studies, immune markers have been compared in subjects with and without IBS without taking into account the severity of the disorders. Liebregts et al. showed a significant association between elevated TNF and IL6 in the blood and the severity of both gastrointestinal symptoms and anxiety [37]. Not having taken into account the severity of the disorder could explain the discrepancies between studies of the immune imbalance in FGID.

The tryptophan-kynurenine metabolism has been associated with a broad range of diseases in the central nervous system, including depression [21, 38]. The tryptophan-kynurenine metabolism is regulated by inflammatory cytokines, and the metabolites are therefore also inflammatory markers. The enzyme indole-2,3-dioxygenase (IDO), which is central in the metabolism of tryptophan, is regulated mainly by INF-*γ* and also by IL-10 and TNF [38]. It is likely that the high tryptophan and kynurenine values that were seen in the CSF in subjects with IBS were associated with the central immune activation. The tryptophan-serotonin-kynurenine pathway has been attributed to be of great importance for the pathophysiology of IBS [19, 20, 39]. A high K/T ratio due to increased catabolism of tryptophan through the kynurenine pathway is a marker of inflammation and has been associated with IBS severity and proposed as a biomarker for IBS [39, 40]. This study did not support these findings.

The associations between the central immune activation, neuroimaging, and disorders such as depression, gastrointestinal complaints, anxiety, fatigue, and muscle-skeletal pain deserve further investigations. It is unknown if any of today's treatments for either depression or FGID, be it drugs, psychotherapy or cognitive therapy, have a central immune-modulating effect. In the future, therapy aiming at the reduction of the central immune activation might be an alternative for a subgroup of subjects with severe gastrointestinal comorbidity.

### 4.2. Strengths and Limitations

The study was designed to compare immune markers including tryptophan and kynurenine in subjects with depression and unexplained neurological symptoms. These subjects have a high prevalence of comorbidity including FGID, which makes them suitable for the study of FGID. The groups were well balanced except for the degree of depression. The limitation related to the inclusion of two different groups of subjects was minimised by adjusting the analyses for the groups and degree of depression.

The inclusion of a healthy control group or a group with isolated FGID would have strengthened the study design. However, to perform a spinal puncture to collect the CSF in healthy volunteers was judged as unethical and would probably not have been approved by the ethics committee.

Despite the limited number of participants, the main results were convincing and highly statistically significant.

The high number of variables used in the analyses of associations with IBS and IBS-SSS without correcting for multiple analyses has increased the probability of type I errors. It strengthens the credibility of the results that the inflammatory markers most often mentioned in relation to IBS (TNF and IL-10) were the only statistically significant inflammatory predictors in this study.

## 5. Conclusions

In this study, high TNF and low IL-10 levels in the CSF were associated with the perceived severity of FGID, and high levels of tryptophan and kynurenine and low levels of IL-10 in the CSF were associated with IBS. The findings might indicate that the gastrointestinal complaints are associated with a proinflammatory central immune activation and an abnormal tryptophan metabolism which could cause and maintain the chronicity of the disorder by a central and long-lasting sensitisation that exaggerates normal and abnormal visceral stimuli.

## Figures and Tables

**Table 1:** Characteristics of the participants in the two groups with comparisons between the groups.

Variables (number of subjects in the analyses if less than 66)	Neurological group *n* = 19	Depression group *n* = 47	Statistics *p* value
Female	12/19 (63%)	25/47 (53%)	0.586^∗^
Age (years)	47 (14)	45 (14)	0.506
BDI-II	6.5 (6.7)	30.4 (12.6)	**<0.001**
IBS	6/19 (32%)	27/47 (57%)	0.102^∗^
IBS-SSS (*n* = 65)	113 (88)	200 (103)	**0.003**
INF-*γ* serum (pg/ml)	63.1 (29.2; 83.0)	54.7 (29.2; 86.4)	**0.025** ^∗∗^
IL-1ra serum (pg/ml)	180 (117; 268)	161 (94; 1825)	**0.027** ^∗∗^
IL-10 serum (pg/ml)	4.05 (2.10; 8.55)	2.99 (0.69; 12.72)	**0.040** ^∗∗^
TNF serum (pg/ml)	30.9 (4.5)	29.8 (5.5)	0.459
TNF/IL-10 ratio serum	7.24 (3.80; 16.19)	9.77 (2.99; 31.88)	**0.029** ^∗∗^
Exotaxin-CSF (pg/ml) (*n* = 62)	2.30 (0.99; 27.79)	1.44 (0.53; 57.73)	**0.042** ^∗∗^
IL-10-CSF (pg/ml) (*n* = 62)	1.64 (0.36)	1.53 (0.32)	0.251
TNF-CSF (pg/ml) (*n* = 62)	1.60 (0.96)	1.43 (0.80)	0.470
TNF/IL-10 ratio CSF (*n* = 62)	1.0 (0.7)	1.0 (0.5)	0.731
Kynurenine serum (*μ*M) (*n* = 62)	2.00 (0.58)	2.17 (0.53)	0.286
Tryptophan serum (*μ*M) (*n* = 62)	56.5 (11.1)	58.1 (12.4)	0.640
K/T ratio × 1000 serum (*n* = 62)	36.1 (9.7)	38.2 (10.1)	0.447
Kynurenine CSF (*μ*M) (*n* = 62)	0.055 (0.037; 0.097)	0.057 (0.032; 0.148)	0.572^∗∗^
Tryptophan CSF (*μ*M) (*n* = 62)	2.12 (1.56; 6.23)	2.07 (1.36; 144.50)	0.708^∗∗^
K/T ratio × 1000 CSF (*n* = 62)	26.4 (7.0)	27.1 (12.2)	0.823
Propionic acid faeces (mmol/l) (*n* = 50)	1.77 (1.11)	1.57 (1.33)	0.582
Butyric acid faeces (mmol/l) (*n* = 50)	2.02 (1.63)	1.40 (1.24)	0.142
PmB in faeces (mmol/l) (*n* = 50)	−0.25 (0.76)	0.16 (0.53)	**0.031**

The results are given as number and proportion (%), mean with standard deviation, and median with range. Fisher's exact test (marked with ∗), *t*-test, and Mann–Whitney *U* test (∗∗) were used for the comparisons between the groups. BDI-II: Beck Depression Inventory-II; IBS: irritable bowel syndrome; IBS-SSS: irritable bowel syndrome symptom severity score; INF-*γ* serum: interferon gamma in serum; IL-10-CSF: interleukin 10 in the cerebrospinal fluid; TNF serum and TNF-CSF: tumor necrosis factor in serum and tumor necrosis factor in the cerebrospinal fluid; PmB: propionic acid minus butyric acid; K/T ratio × 1000: kynurenine/tryptophan ratio × 1000.

**Table 2:** Predictors of IBS. The covariates in the analyses were the groups (neurological/depression), depression (BDI-II), and one by one of the other variables.

Group		BDI-II		Other variables		
OR (95% CI)	*p* value	OR (95% CI)	*p* value		OR (95% CI)	*p* value
2.93 (0.95; 9.03)	0.062					
0.66 (0.13; 3.31)	0.617	1.07 (1.02; 1.12)	**0.012**			
0.58 (0.11; 3.00)	0.511	1.07 (1.02; 1.13)	**0.009**	Gender (male)	1.71 (0.58;5.09)	0.333
0.64 (0.13; 3.24)	0.590	1.07 (1.02; 1.13)	**0.011**	Age (years)	1.00 (0.97;1.05)	0.723
0.69 (0.14; 3.46)	0.651	1.07 (1.02; 1.13)	**0.009**	IL-10 serum (pg/ml)	1.12 (0.88;1.43)	0.357
0.75 (0.15; 3.79)	0.725	1.07 (1.02; 1.12)	**0.013**	TNF serum (pg/ml)	1.07 (0.95; 1.20)	0.246
0.73 (0.14; 3.72)	0.702	1.07 (1.02; 1.12)	**0.011**	TNF/IL-10 ratio serum	0.97 (0.87; 1.08)	0.572
0.59 (0.10; 3.46)	0.561	1.07 (1.01; 1.13)	**0.020**	IL-10-CSF (pg/ml)	0.15 (0.02; 0.97)	**0.047**
0.56 (0.10; 3.11)	0.509	1.08 (1.02; 1.13)	**0.008**	TNF-CSF (pg/ml)	1.10 (0.57; 2.11)	0.776
0.59 (0.11; 3.33)	0.550	1.07 (1.02; 1.13)	**0.011**	TNF/IL-10 ratio CSF	1.34 (0.52; 3.48)	0.548
0.49 (0.09; 2.76)	0.418	1.08 (1.02; 1.14)	**0.007**	Kynurenine serum	1.52 (0.54; 4.30)	0.429
0.46 (0.08; 2.61)	0.384	1.08 (1.02; 1.15)	**0.005**	Tryptophan serum	1.05 (0.99; 1.11)	0.107
0.56 (0.10; 3.05)	0.500	1.08 (1.02; 1.14)	**0.008**	K/T × 1000 serum	0.99 (0.94; 1.05)	0.754
0.55 (0.10; 2.97)	0.482	1.08 (1.02; 1.14)	**0.008**	Kynurenine CSF	>1.00	**0.019** ^∗^
0.53 (0.09; 3.19)	0.489	1.08 (1.02; 1.14)	**0.009**	Tryptophan CSF	>1.00	**0.048** ^∗^
0.54 (0.10; 2.96)	0.480	1.08 (1.02; 1.14)	**0.008**	K/T × 1000 CSF	1.00 (0.95; 1.05)	0.844
0.32 (0.04; 2.29)	0.254	1.13 (1.04; 1.22)	**0.004**	Propionic acid faeces	1.90 (1.04; 3.46)	**0.036**
0.35 (0.05; 2.34)	0.280	1.11 (1.03; 1.20)	**0.007**	Butyric acid faeces	1.06 (0.67; 1.69)	0.800
0.08 (0.01; 1.10)	0.059	1.17 (1.05; 1.31)	**0.006**	PmB faeces	6992 (10; 4,793,010)	**0.008**

^∗^Fractional polynomial logistic regression. BDI-II: Beck Depression Inventory-II; IL-10 serum: interleukin 10 in the serum; TNF serum: tumor necrosis factor in serum; IL-10-CSF: interleukin 10 in the cerebrospinal fluid; TNF-CSF: tumor necrosis factor in the cerebrospinal fluid; PmB: propionic acid minus butyric acid in faeces; K/T ratio × 1000: kynurenine/tryptophan ratio × 1000.

**Table 3:** Predictors of IBS-SSS. The covariates in the analyses were the groups (neurological/depression), depression (BDI-II), and one by one of the other variables.

Group		BDI-II		Other variables		
B (95% CI)	*p* value	B (95% CI)	*p* value		B (95% CI)	*p* value
86 (31; 141)	**0.003**					
28 (−45; 102)	0.444	2.4 (0.3; 4.6)	**0.027**			
23 (−51; 98)	0.533	2.6 (0.4; 4.7)	**0.021**	Gender (male)	24 (−24; 73)	0.317
23 (−51; 98)	0.529	2.8 (0.6; 5.0)	**0.015**	Age (years)	1.0 (−0.7; 2.8)	0.252
28 (−46; 102)	0.454	2.4 (0.2; 4.6)	**0.031**	IL-10 serum (pg/ml)	−1.5 (−12.6; 9.6)	0.787
30 (−45; 105)	0.429	2.4 (0.3; 4.6)	**0.029**	TNF serum (pg/ml)	0.8 (−3.9; 5.4)	0.745
24 (−51; 99)	0.526	2.4 (0.3; 4.6)	**0.028**	TNF/IL-10 ratio serum	1.8 (−3.2; 6.8)	0.480
23 (−54; 99)	0.551	2.3 (0.0; 4.5)	**0.048**	IL-10-CSF (pg/ml)	−69 (−146; 9)	0.084
30 (−46; 106)	0.432	2.5 (0.3; 4.7)	**0.024**	TNF-CSF (pg/ml)	32 (3; 61)	**0.030**
33 (−42; 108)	0.380	2.2 (0.0; 4.4)	**0.046**	TNF/IL-10 ratio CSF	55 (13; 97)	**0.012**
21 (−58; 101)	0.595	2.7 (0.5; 2.0)	**0.020**	Kynurenine serum	0.5 (−46.2; 47.1)	0.984
24 (−53; 102)	0.530	2.6 (0.4; 4.9)	**0.021**	Tryptophan serum	−1.4 (−3.5; 0.7)	0.194
17 (−61; 95)	0.668	2.8 (0.5; 5.0)	**0.017**	K/T × 1000 serum	1.3 (−1.2; 3.8)	0.304
34 (−44; 111)	0.387	2.4 (0.2; 4.6)	**0.034**	Kynurenine CSF (pg/ml)	>1	0.062^∗^
22 (−56; 100)	0.577	2.8 (0.6; 5.0)	**0.015**	Tryptophan CSF (pg/ml)	>1	0.150^∗^
21 (−57; 99)	0.589	2.7 (0.5; 5.0)	**0.019**	K/T × 1000 CSF	−0.3 (−2.6; 2.0)	0.797
40 (−42; 122)	0.332	2.6 (−0.2; 5.3)	0.065	Propionic acid faeces (mmol/l)	1.9 (−20.7; 24.4)	0.869
35 (−47; 118)	0.390	2.5 (−0.2; 5.2)	0.070	Butyric acid faeces (mmol/l)	−7.2 (−27.7; 13.2)	0.481
22 (−58; 102)	0.580	2.4 (−0.1; 5.1)	0.063	PmB faeces (mmol/l)	44 (−0.4; 87.6)	0.052

^∗^Fractional polynomial linear regression. BDI-II: Beck Depression Inventory-II; IL-10 serum: interleukin 10 in the serum; TNF serum: tumor necrosis factor in serum; IL-10-CSF: interleukin 10 in the cerebrospinal fluid; TNF-CSF: tumor necrosis factor in the cerebrospinal fluid; PmB: propionic acid minus butyric acid in faeces; K/T ratio × 1000: kynurenine/tryptophan ratio × 1000.

**Table 4:** Predictors of IBS and IBS-SSS. The covariates in the logistic and linear regression analyses were group, BDI-II, and IL-10 and TNF in serum and CSF. The results are given as OR (95% CI) and *p* value for the logistic regression analyses and as B (95% CI) and *p* value and partial correlation for the linear regression analyses.

Dependent variables (number of subjects)	Independent variables										
	Group		BDI-II			TNF			IL-10		
	B (95% CI) OR (95% CI)	*p* value	B (95% CI) OR (95% CI)	*p* value	Partial corr.	B (95% CI) OR (95% CI)	*p* value	Partial corr.	B (95% CI) OR (95% CI)	*p* value	Partial corr.
						TNF serum			IL-10 serum		
IBS	OR: 0.72 (0.1; 3.8)	0.717	OR: 1.07 (1.01;1.12)	**0.013**		OR: 1.06 (0.92; 1.22)	0.428		OR: 1.04 (0.77; 1.41)	0.807	
IBS-SSS (65)	B: 30 (− 45; 106)	0.418	B: 2.3 (0.1; 4.5)	**0.042**	0.260	B: 1.8 (−4.1; 7.6)	0.543	0.079	B: −4.1 (−18.2; 9.9)	0.561	−0.075
						TNF-CSF			IL-10 CSF		
IBS (62)	OR: 0.63 (0.1; 3.7)	0.609	OR: 1.07 (1.00;1.13)	**0.023**		OR: 1.25 (0.63; 2.47)	0.528		OR: 0.13 (0.02; 0.92)	**0.04**	
IBS-SSS (61)	B: 33 (−39, 107)	0.360	B: 1.9 (−0.2; 4.1)	0.080	0.232	B: 38 (10; 67)	**0.009**	0.341	B: −88 (−163, −13)	**0.023**	−0.299

BDI-II: Beck Depression Inventory-II; IL-10 serum: interleukin 10 in the serum; TNF serum: tumor necrosis factor in serum; IL-10-CSF: interleukin 10 in the cerebrospinal fluid; TNF-CSF: tumor necrosis factor in the cerebrospinal fluid.
